# Reconstructing historical 3D city models

**DOI:** 10.1007/s44212-022-00011-3

**Published:** 2022-10-21

**Authors:** Camille Morlighem, Anna Labetski, Hugo Ledoux

**Affiliations:** 1 Department of Geography, University of Namur, Namur, Belgium; 2 ILEE, University of Namur, Namur, Belgium; 3 3D geoinformation research group, Delft University of Technology, Delft, The Netherlands

**Keywords:** 3D city models, Historical maps, Map alignment, Procedural modelling

## Abstract

Historical maps are increasingly used for studying how cities have evolved over time, and their applications are multiple: understanding past outbreaks, urban morphology, economy, etc. However, these maps are usually scans of older paper maps, and they are therefore restricted to two dimensions. We investigate in this paper how historical maps can be ‘augmented’ with the third dimension so that buildings have heights, volumes, and roof shapes. The resulting 3D city models, also known as *digital twins*, have several benefits in practice since it is known that some spatial analyses are only possible in 3D: visibility studies, wind flow analyses, population estimation, etc. At this moment, reconstructing historical models is (mostly) a manual and very time-consuming operation, and it is plagued by inaccuracies in the 2D maps. In this paper, we present a new methodology to reconstruct 3D buildings from historical maps, we developed it with the aim of automating the process as much as possible, and we discuss the engineering decisions we made when implementing it. Our methodology uses extra datasets for height extraction, reuses the 3D models of buildings that still exist, and infers other buildings with procedural modelling. We have implemented and tested our methodology with real-world historical maps of European cities for different times between 1700 and 2000.

## Introduction

**Fig. 1:**
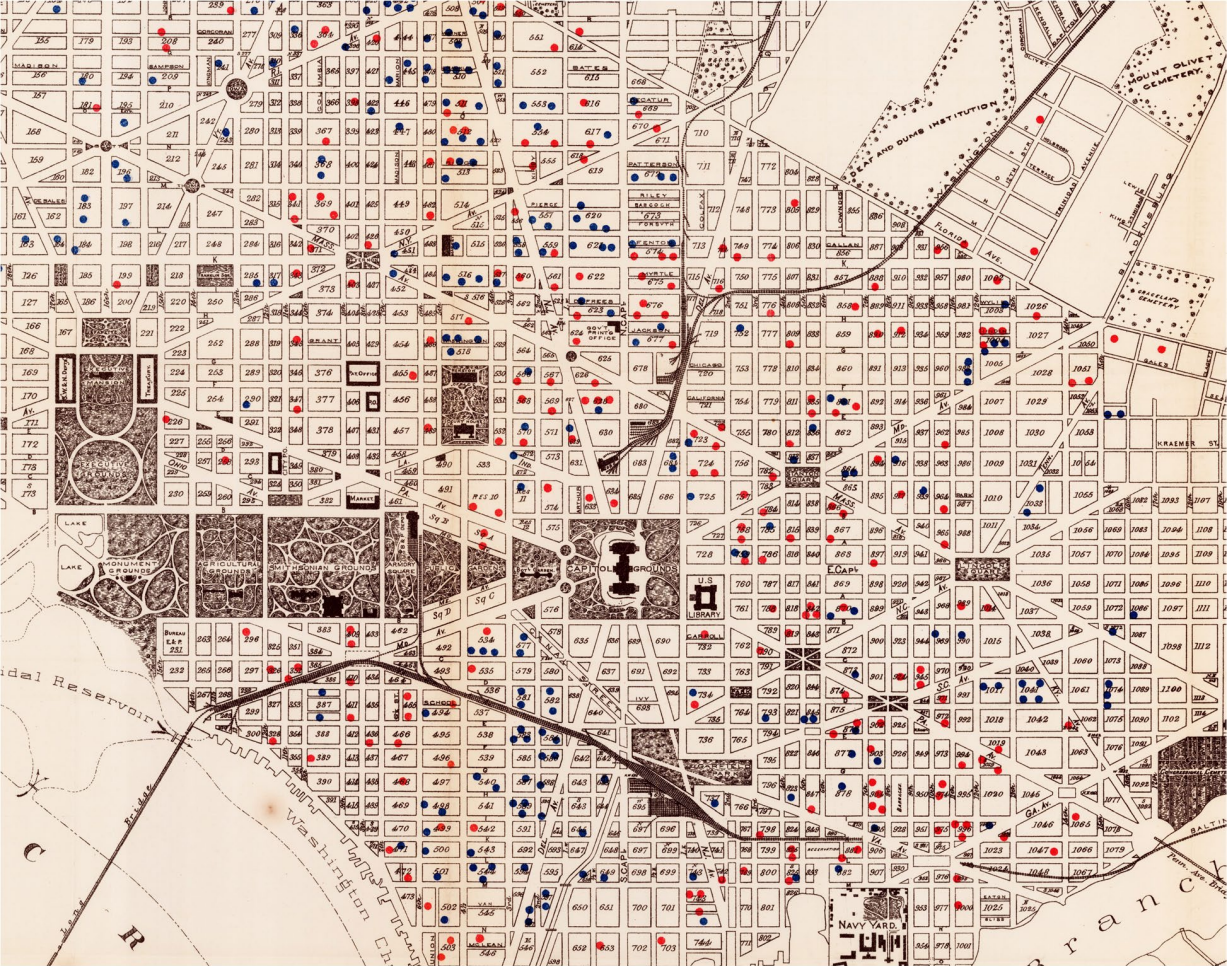
Historical map showing the tuberculosis deaths in Washington, D.C. in 1900–1901. Tuberculosis deaths are represented by blue and red spots. Available at https://lib.msu.edu/

Historical maps are often the only information available about the pre-satellite and digital map era; they contain information that cannot be found elsewhere (Liu et al., 2019). They are therefore an invaluable source of information in various domains and for various use cases. They can help us understand the current configuration of a landscape or of a city as this configuration relies on the past changes that took place (Nobajas & Nadal, 2015), they have been used for understanding urban studies and the economy (Balletti & Guerra, 2016), and they can help understand past outbreaks of diseases. For example, Fig. 1 shows the spatial distribution of tuberculosis deaths in Washington, D.C. in 1900.

Three-dimensional (3D) city models, also known as *digital twins* (Ketzler et al., 2020), digital representations of the objects in a city (e.g. buildings, trees, and roads), increase the level of understanding of what a city looks like (instead of reducing it to a 2D plane), such that we can observe it from different viewpoints and better catch the real size of objects, and therefore they allow us to perform spatial analyses and simulations that are not possible in 2D. Biljecki et al. (2015) discuss 29 use cases for 3D city models, and report more than 100 applications where they are nowadays used. These use cases are varied: solar potential, tracking of pollutants in a city, visibility queries, etc.

**Fig. 2:**
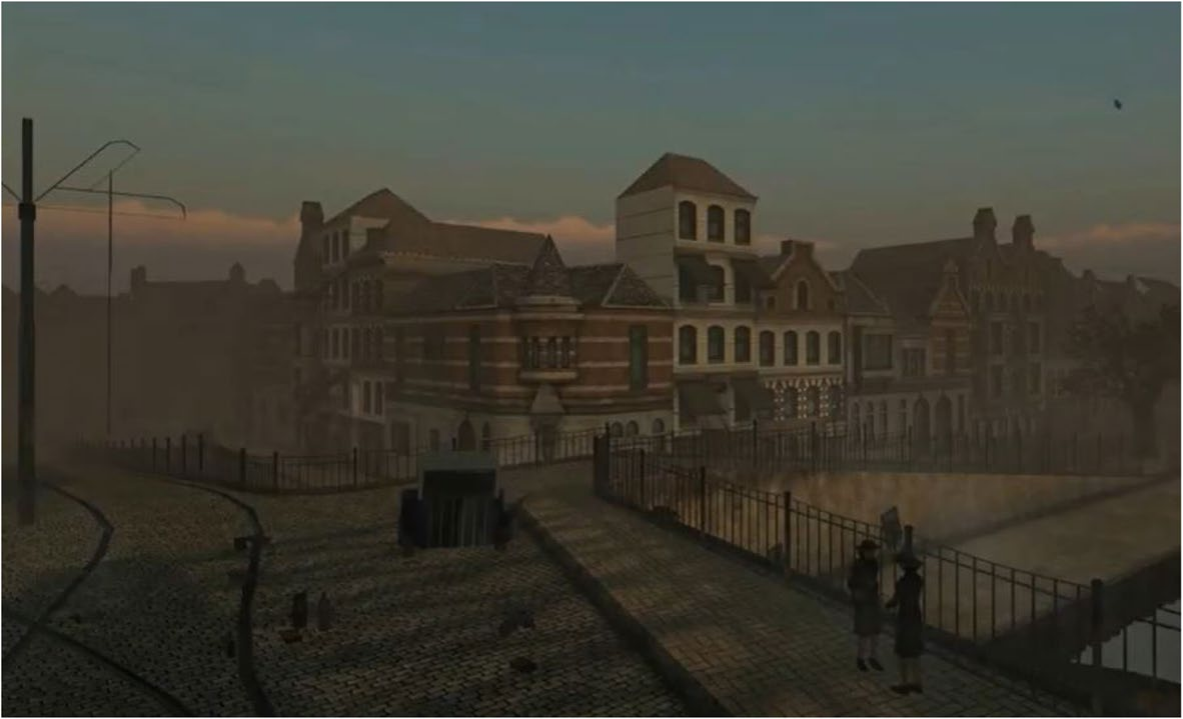
Historical 3D city model of Rotterdam in 1940

**Fig. 3:**
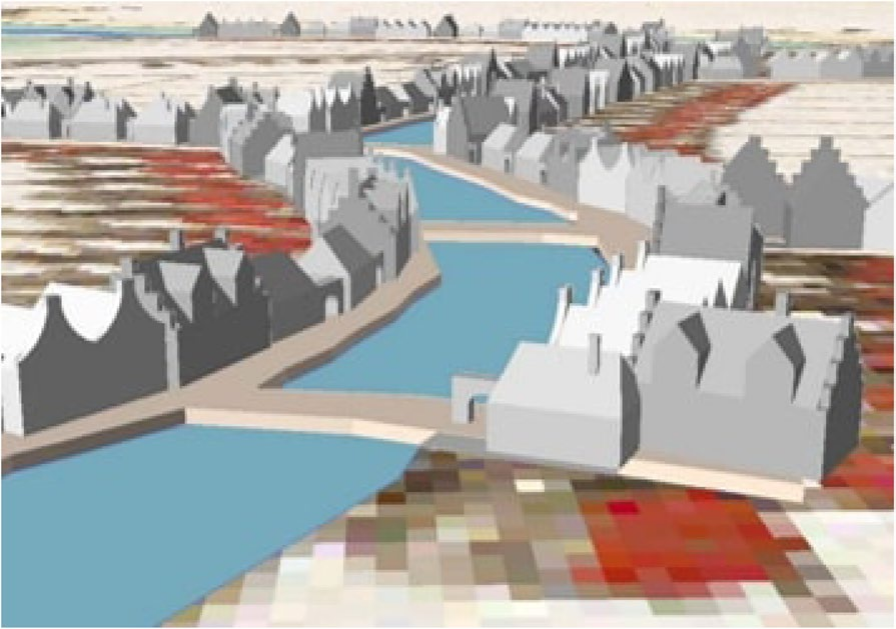
Simple historical 3D city model of Cruquius in the 17^th^ century. Figure from de Boer (2010)

We investigate in this paper *historical* 3D city models, which we refer to as 3D models at different times containing buildings and/or other features in the built environment. Some of these models can be highly detailed and realistic, see for instance Frischer et al. (2008) or the model of the City of Rotterdam in 1940 (one part is shown in Fig. 2). Such models greatly help with the preservation and communication of the historical heritage, allowing for the widespread diffusion of historical sites over the Internet or in museums for cultural and educational purposes (Kersten et al., 2012, Balletti & Guerra, 2016). A major downside of these models is that they can only be reconstructed manually, and therefore they require lots of time, labour, and money. As an example, for the models of Rotterdam (at three epochs), it required ten people working full-time over eight months. However, other studies have focused on reconstructing less detailed historical 3D city models, see Fig. 3 for an example in the Netherlands from de Boer (2010).

These simpler models can also find interesting applications in practice. First, they come as a good mean to give a context to more specific reconstructions. For instance, they can be used as a basis for reconstructing more detailed historical 3D city models such as Rotterdam’s as they already provide simple 3D objects, which can be further refined. There are already methods that automate parts of this process, see for instance Zhang et al. (2019) and Wang (2022). Second, they can be used to estimate the population of a city at times when demographic data are not available. Such information can be further useful for another major use case: historic disease mapping. Raising Fig. 1 to the third dimension could help recover information about the population distribution and thus give additional insights into how and where the disease spread in the city in 1900 (knowing that tuberculosis is a contagious disease). Existing research on the correlation between plague deaths and the socioeconomic status of households in Dijon (Galanaud et al., 2015) could be extended by studying the influence of population density derived from a simple historical 3D city model. Finally, simple historical 3D city models could also be used for visibility analyses or to perform past microclimate analyses (Biljecki et al., 2015), in computational fluid dynamics (Pađen et al., 2022), and recover information such as the past ground temperature.

The main bottleneck with the use of these simple historical 3D city models in practice is that their reconstruction is a time-consuming operation that still nowadays requires several manual operations. Indeed, data imperfection in historical maps makes the reconstruction particularly challenging (Kersten et al., 2012). Historical maps are usually affected by geometric and chronometric inaccuracies because of the way they were created back in the day, using triangulation networks, but also because they were often influenced by the subjectivity of the cartographers (de Boer, 2010). Furthermore, it is rare to obtain a complete set of historical data covering the whole study area (Herrault et al., 2013a).

We focus in this paper on *automating as much as possible* the reconstruction of historical 3D city models, and we focus on arguably the most important aspect in cities: buildings. We present a new methodology that allows us to reconstruct *plausible* building models of European cities, i.e. we recognise that we are limited by the amount of historical information available, and we make educated guesses and assumptions in order to infer 3D models that most likely match reality at a given moment in time. As further explained, our methodology has three main steps and uses as input georeferenced scanned historical maps of an area, extra datasets to extract the height (aerial point cloud LiDAR dataset (Mallet & Bretar, 2009), and existing 3D models of the present day. We reuse the 3D models of buildings that still exist, and we reconstruct other buildings by inferring their shapes and roofs with procedural modelling. We reconstruct buildings with hip or gable roofs, among others, those are referred to as LoD2 in the common GIS community and are between LoD1 with simple flat roofs and LoD3 with more details and additional building installations (e.g. windows); see Biljecki et al. (2016b) for details. We implemented our methodology with open source code, and we tested it with real-world historical maps of Delft (the Netherlands) and Brussels (Belgium) for different time periods between 1700 and 2000. All datasets and codes used are publicly available, so our results can be easily replicated and adapted to other cities across Europe^1^.

## Related work

The initial attempts at modelling in 3D past landscapes or cities date back to the 1980s, but historical 3D modelling was only truly made possible from the 1990s with the evolution of 3D GIS and CAD software (de Boer, 2010). One example of pioneer research was the Rome Reborn Project, which started in 1997 and aimed at reconstructing—manually—historical 3D city models showing the evolution of Rome from the Bronze Age to the Middle Ages (Frischer et al., 2008).

In recent years, there has been increasing research on reconstructing historical 3D city models from historical sources. Nobajas and Nadal (2015) manually digitised historical cadastral plans to extract building footprints and further overlay them with a generic 3D building model. Another solution was proposed by Kersten et al. (2012) to reconstruct a historical 4D city model (i.e. a 3D city model with time as fourth dimension) from a wooden model of the city of interest and historical maps manually digitised. The complete reconstruction of the final model required 800 hours in total, with half of the time spent in manual processing steps. Similarly, Balletti and Guerra (2016) reconstructed a historical 4D city model from a collection of historical documents. Their methodology is based on the combination of database archiving for storing these documents and GISs for the 3D modelling of the city.

Although these methods produce accurate and promising results, they rely strongly on manual processing steps. Some attempts have been made for automating the process. de Boer (2010) proposed a GIS-based method relying on histogram thresholding for extracting building footprints from historical maps. They made use of generic 3D building models to overlay over these building footprints. However, histogram thresholding restricts the use of their methodology to good quality historical maps with high colour contrasts. Other research has been conducted for automating the reconstruction of historical 3D city models of the Edo era in Japan. Suzuki and Chikatsu (2003) used historical paintings and remaining historic houses of the Edo period to create 3D parametric models of the different types of houses and combined them with building footprints extracted from historical maps. However, their approach only makes it possible to reconstruct 3D buildings with rectangular footprints.

As a result, when existing methods do not rely on manual processing, they require as input certain types of historical maps or only allow to reconstruct certain types of buildings (Liu et al., 2019). Some research suggests that it is not possible to find a unique methodology working for any collection of historical maps, as they all have their own symbology, colours, hues, and contrasts (Sun et al., 2020). In this study, we automated as much as possible the workflow going from the processing of the historical maps to the generation of the final historical 3D city model, working on different historical map collections and allowing diversity in the type of buildings (i.e. regarding the roof type and the shape of the building footprints). We summarise all the methods we investigated and tried in Table 1.

**Table 1:** Overview of the different methods used, implemented and compared to develop a building plot extraction workflow

	**Method**	**Overview of the methodology**
Arteaga (2013)	Building footprint extraction	This methodology relies on a thresholding step in GIMP to produce a black and white image from the historical map. Polygons are vectorised and generalised based on an $\alpha$-shape operator and building polygons are identified based on their average colour.
Drolias and Tziokas (2020)	Building footprint extraction	The input historical map is manually reclassified in QGIS to generate a binary raster. After some cleaning steps, building footprints are vectorised and generalised using a Douglas-Peucker (DP) algorithm.
de Boer (2010)	Building footprint extraction	A Delaunay triangulation is first built in ArcGIS from all building pixels manually selected. After removing large triangles, adjacent ones are merged to recreate building footprints.
Gobbi et al. (2019)	Historical map digitalisation	This method, developed in GRASS GIS, relies on performing a segmentation and OBIA classification of the input historical maps. It also provides a method for cleaning historical maps from their textual information.
Henderson et al. (2009)	Historical map digitalisation	Unsupervised classification algorithms are used for automatically segmenting raster maps, such as the expectation maximisation algorithm and the K-means clustering algorithm.
Herrault et al. (2013b)	Historical map digitalisation	This method relies on preprocessing steps based on dilation and smoothing operators to remove textual features, lines and symbols from input historical maps before digitalising them with K-means clustering algorithms.
scikit-learn supervised algorithms	Historical map digitalisation	Supervised classification algorithms such as random forest can be used to digitalise an input historical map using training data.
Commandeur (2012)	Building footprint generalisation	The algorithm merges boundary line segments which are nearly parallel based on distance and angle thresholds between line partitions.
Douglas and Peucker (1973)	Building footprint generalisation	The DP algorithm iteratively keeps points within a tolerance distance from the polyline being generalised until it reaches the defined number of remaining vertices.
CGAL simplification algorithm	Building footprint generalisation	This algorithm generalises a polyline and preserves its topology by using a tolerance distance or by keeping a defined percentage or number of vertices from the total number of vertices.

## Our automated methodology

**Fig. 4:**
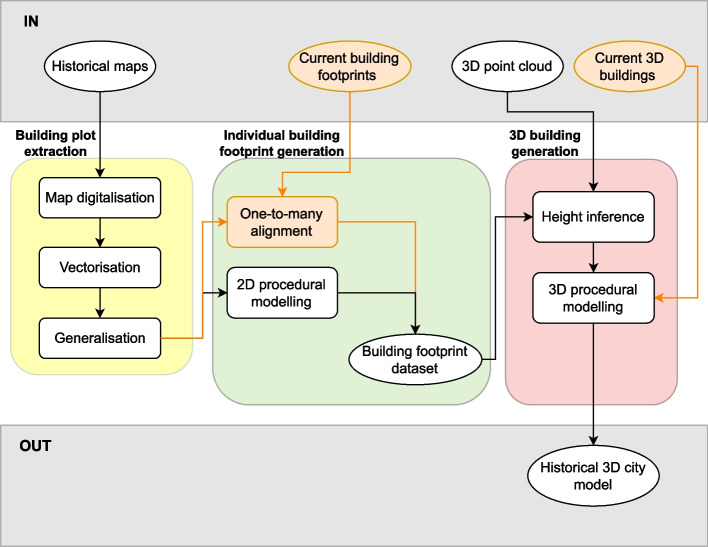
General methodology workflow. Rectangular boxes represent methodology steps and oval boxes represent datasets. Optional datasets and steps relying on data availability are depicted in orange

The methodology we propose to reconstruct the 3D model of a city at different times consists of three main steps: (1) the processing of the historical maps to extract the building plots, (2) the subdivision of these building plots into individual building footprints and (3) the reconstruction of 3D buildings. The only user interventions that are needed to implement it are the generation of training and testing datasets for the building plot extraction step—this is further detailed in Section 3.1—and the passing of user-defined parameter values (e.g. facade length and depth). The whole workflow is shown in Fig. 4 and we further describe it in the following sections.

### Step 1: Building plot extraction

**Fig. 5:**
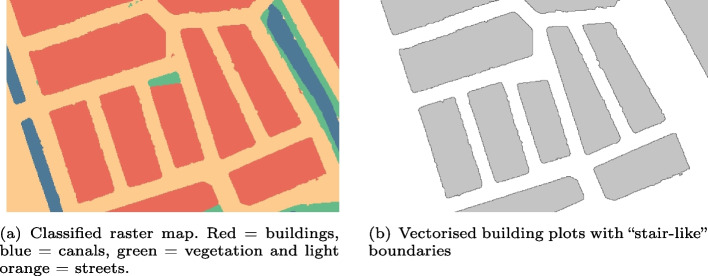
Example of vectorised building plots extracted from a classified raster map

The first step of the workflow is the extraction and vectorisation of the building plots from the input historical map. We started by digitalising the historical map using the method of Gobbi et al. (2019) (as implemented in GRASS GIS). Their method consists in segmenting the historical map into patches sharing colour and shape properties. The segmented map is further classified into different categories of land use using an Object-Based Image Analysis (OBIA) classification. This classification requires training data. To facilitate their creation, the method of Gobbi et al. (2019) makes use of training data points. Points are generated in the geographic extent covering the historical map, and they are manually assigned the land use class in which they are located. With a point-in-polygon procedure, the labels of the training points are transferred to the segments in which they lie, obtaining training segments. The digitalisation ends by applying a low-pass filter on the classified map to remove textual features and symbols, which could, if not removed, disrupt the vectorisation of the building plots over which they are overlaid. Once the historical map has been digitalised, the building plots are vectorised using the polygonize tool from GDAL. As the vectorisation procedure creates polygons with ‘stair-like’ boundaries (Fig. 5), we smooth their contour a first time with the generalisation algorithm of Arteaga (2013) and a second time with the algorithm of Commandeur (2012) (see Table 1).

**Fig. 6:**
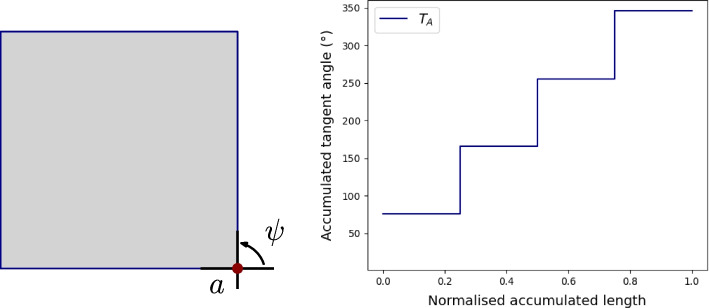
Representation of a polygon (left) using its turning function (right)

In order to assess the performance of the building plot extraction, we create a testing set by manually adding a point in each building plot on the historical map. With a point-in-polygon procedure, we are able to count the number of building plots that have been properly classified and compute the precision, the recall and the F-score. In addition, in order to assess whether the geometries of the building plots were accurately recovered, a subset of them is manually digitised from the historical map and compared with the corresponding building plots extracted with our methodology. Two metrics are used to measure shape similarity. The first metric compares two shapes by computing the difference between their turning functions. When turning around a shape, it is possible to compute at each vertex the counter-clockwise angle between the two adjacent edges (Fan et al., 2014), see Fig. 6, where $\psi$ represents the counter-clockwise angle at vertex *a*. The turning function expresses how the accumulated tangent angle changes with the normalised accumulated edge length (Fig. 6). As each shape is uniquely described by its turning function, the smaller the difference between the turning functions of two shapes, the more the shapes are similar (Fan et al., 2014; Arvin et al., 1991). This metric is scale and rotation invariant, but it is sensitive to noise, hence it is used to assess whether the angles and the contour of the building plots were properly extracted. To further check whether the building plots were properly extracted regarding their size and orientation, we use the shape similarity metric of Samal et al. (2004), where similarity between two shapes is computed as the average percentage of one shape within the buffer area of the second. This measure varies between 0 (the shapes are not even intersecting) and 100 (the shapes are perfectly matching with each other).

### Step 2: Individual building footprint generation

Historical maps do not always depict buildings as individual building footprints. Instead, they might be aggregated into building plots. Before reconstructing 3D buildings, these building plots must be split into individual building footprints. We used two processes for this purpose: map alignment and 2D procedural modelling.

#### Map alignment

**Fig. 7:**
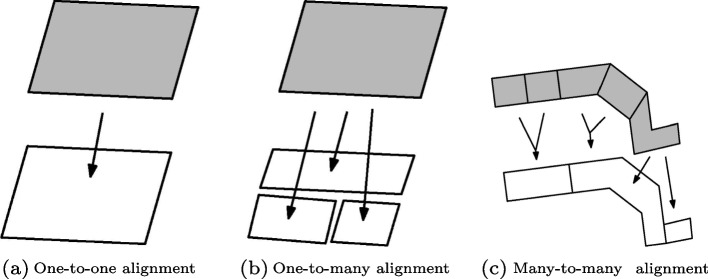
Types of map alignment. Figure modified from Xavier et al. (2016)

Historic cities sometimes still have very old constructions that are more than a century old. Thus, some buildings that are still present nowadays might be depicted on the historical map. We use the map alignment to identify these buildings so that their current building footprints can be used to subdivide the historical building plots. The goal of map alignment is to identify the geographic features that are present in two or more maps—namely the aligned features (Xavier et al., 2016). Different types of map alignment exist, depending on the number of features to be matched (Fig. 7). In this study, we used a one-to-many alignment to match the extracted building plots with the building footprints from a current dataset of the city of interest. To check whether an historical building plot *a* can be matched with a building footprint *b*, the similarity measure $\sigma (a,b)$ is computed using the overlapping area between the building footprint and the buffered building plot (Eq. 1). The value obtained is compared with a user-defined threshold. From our observations and experiments with several historical maps, we recommend using a threshold of 60%. However, this value can still be adjusted given the accuracy/age of the historical map. When historical maps are affected by strong geometric inaccuracies, buildings might sometimes be shifted from their true location, in which case lowering the threshold will make sure these historic buildings are still matched. Only the building footprints with a year of construction prior to the date at which the historical map was made are used for the map alignment. This must be ensured to avoid cases where historic buildings were completely destroyed and replaced with new constructions. In such cases, the footprints of these new constructions would still be aligned with historical building plots at the same location, even though they have very different construction designs. From this step, building footprints that were found to be aligned with historical building plots are used to subdivide them into individual building footprints.

1 $$\begin{aligned} \sigma (a,b) = \frac{area_\mathrm {overlap}}{\min (area_\mathrm {a}, area_\mathrm {b})} \end{aligned}$$

#### 2D procedural modelling

**Fig. 8:**
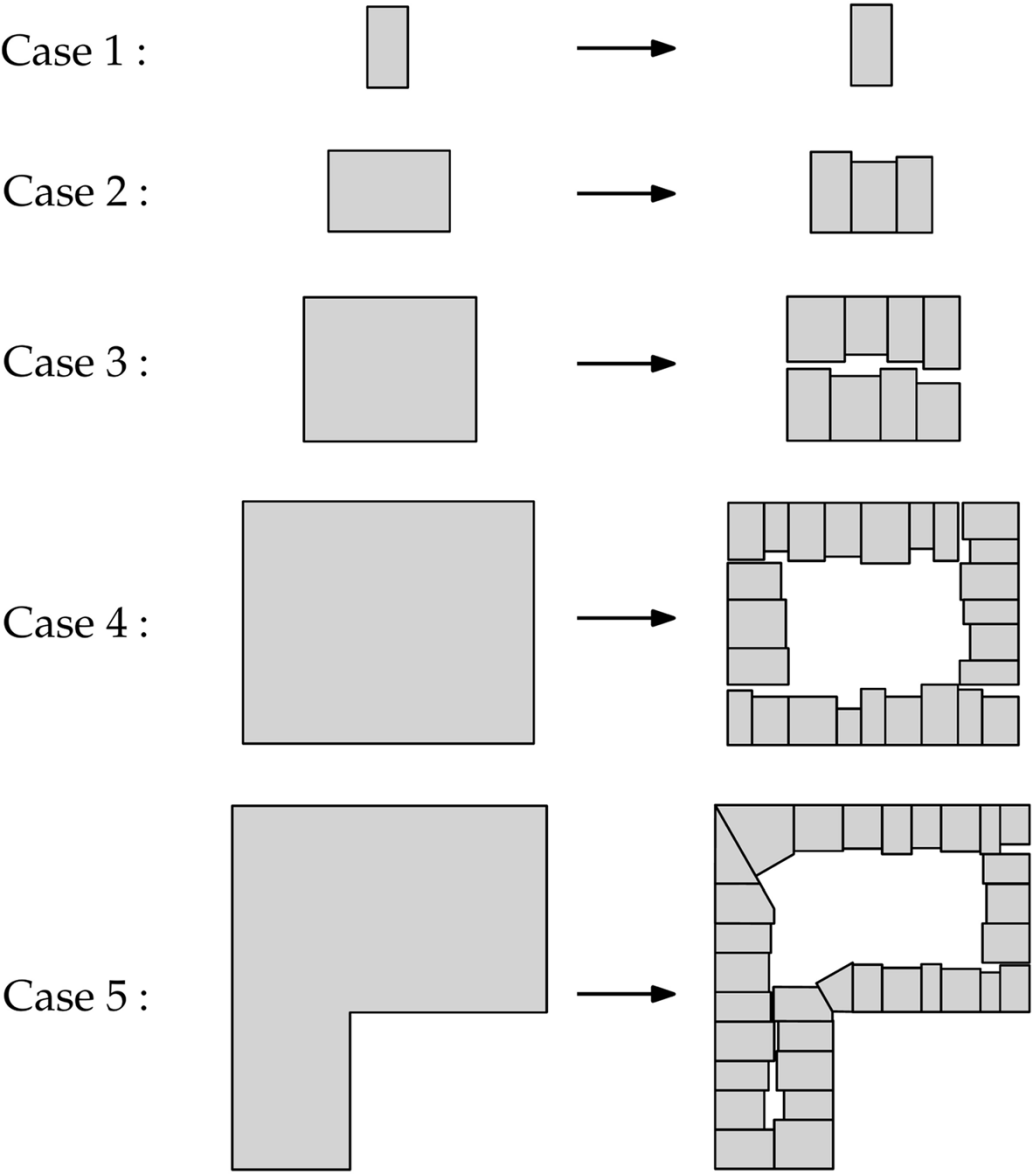
Overview of the five cases used to subdivide a building plot into building footprints using 2D procedural modelling

Although some buildings on the historical map still remain presently, there are also buildings that were demolished and replaced with new constructions over the years. To recover their footprint, we used a process called procedural modelling. In computer graphics, this process is used for reconstructing 2D or 3D objects in an automatic and generative way from a user-defined set of rules. In this study, we used 2D procedural modelling to reconstruct individual building footprints automatically from building plots. We developed five main cases to handle the subdivision of any building plot, based on its size and shape (Fig. 8):

- **Case 1:** The area of the building plot is below a threshold such that the building plot is already a building footprint, and it is kept as is.
- **Case 2:** One single row of building footprints fits inside the building plot, such that the building plot is split into building footprints with variable lengths and depths (based on an original user-defined length and depth) perpendicularly along its longest edge.
- **Case 3:** Two rows of building footprints fit inside the building plot. The building plot is split along its longest median to create two polygons (i.e. the two building rows), which are individually processed using case 2.
- **Case 4:** As the building plot is too big to be split entirely into building footprints, we assume that it contains an interior courtyard or continuous space made of gardens, an arrangement that is common in many European cities. We use the straight skeleton (Held & Palfrader, 2017) of the building plot to create an inward offset polygon inside it and generate building footprints with variable lengths and depths around that offset polygon.
- **Case 5:** The building plot has a non-convex shape. The convex decomposition algorithm of Bayazit (2009) is used to split it into convex polygons, which are further processed individually using case 1 to 4.

The building plots can be subdivided either by combining both processes, or by using only 2D procedural modelling. Using one solution or the other depends on the availability of a current building footprint dataset with specific attributes (i.e. building year of construction), which are needed to implement the map alignment. User-defined building length and depth can either come from the literature, i.e. using standard building depth and length in the region of interest, or they can be based on the average length and depth of the aligned (historic) building footprints if the map alignment was implemented (or the average length and depth measured on current building footprint data). Similarly, for the area threshold (case 1), one can use the minimum building footprint area in the aligned building footprints (or in a current building footprint dataset if the map alignment was not implemented).

The output from this step is a dataset with at most two types of building footprints: the aligned ones and the ones generated with 2D procedural modelling—which we refer to as ‘2PM building footprints’ from this point.

### Step 3: 3D building generation

The last step of the methodology deals with the reconstruction of 3D buildings. The final historical 3D city model is reconstructed by: (i) generating height attributes; (ii) reconstructing 3D buildings with 3D procedural modelling; and (iii) generating a valid CityJSON file (Ledoux, 2018). CityJSON is a simple format to store semantic 3D city models and is an international standard of the Open Geospatial Consortium (OGC).

#### Height inference

The building footprints need to have a ground and a roof height to be reconstructed in 3D. As the aligned building footprints already have such height attributes, only the 2PM building footprints need to be assigned some. For assigning the ground height, we used the method of Dukai et al. (2019), which makes use of a current 3D point cloud made of ground points (a raster digital elevation model, typically available in several countries, could also be used). A point-in-polygon procedure is implemented to find the ground points located in the building footprints. From these ground points, the median height is computed and used as reference ground height. The roof height is assigned to the 2PM building footprints based on their neighbouring aligned building footprints, as these already have height attributes. In our methodology, we make the assumption that the roof height is the median height of the building neighbours (but this could be modified to include some randomness, for instance) (Kersten et al., 2012). If the map alignment was not implemented, this method cannot be used and the roof height is instead randomly generated based on regulated roof heights that were common in the period in which the historical map was made.

#### 3D procedural modelling

Procedural modelling is used to generate automatically (textured) 3D city models from vector data using the *computer generated architecture* (CGA) grammar of Parish & Müller (2001). Their methodology works by reconstructing a crude 3D city model made of flat building blocks using a set of high-priority rules. Then, low-priority rules are used to add details and generate more complex buildings. In our methodology, we use the *Computer Generated Architecture for Blender* (BCGA) addon, which is an open source implementation of the CGA grammar. We use three types of rules:

- **Rule 1:** extrudes the building footprint at its assigned roof height;
- **Rule 2:** decomposes the resulting 3D shape into different parts (top, front, and side faces) and,
- **Rule 3:** generates a roof above the top face of the 3D shape.

**Fig. 9:**
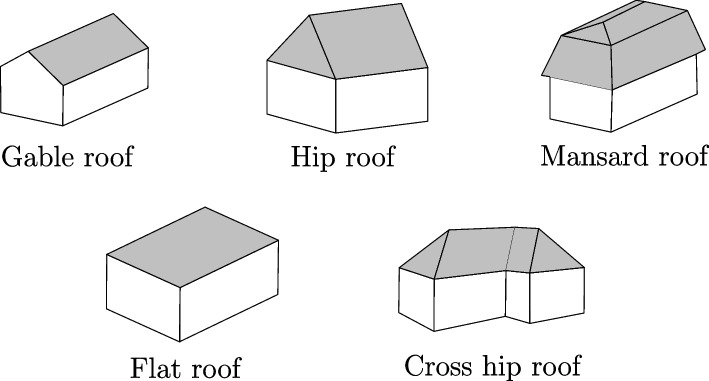
Common roof types

**Fig. 10:**
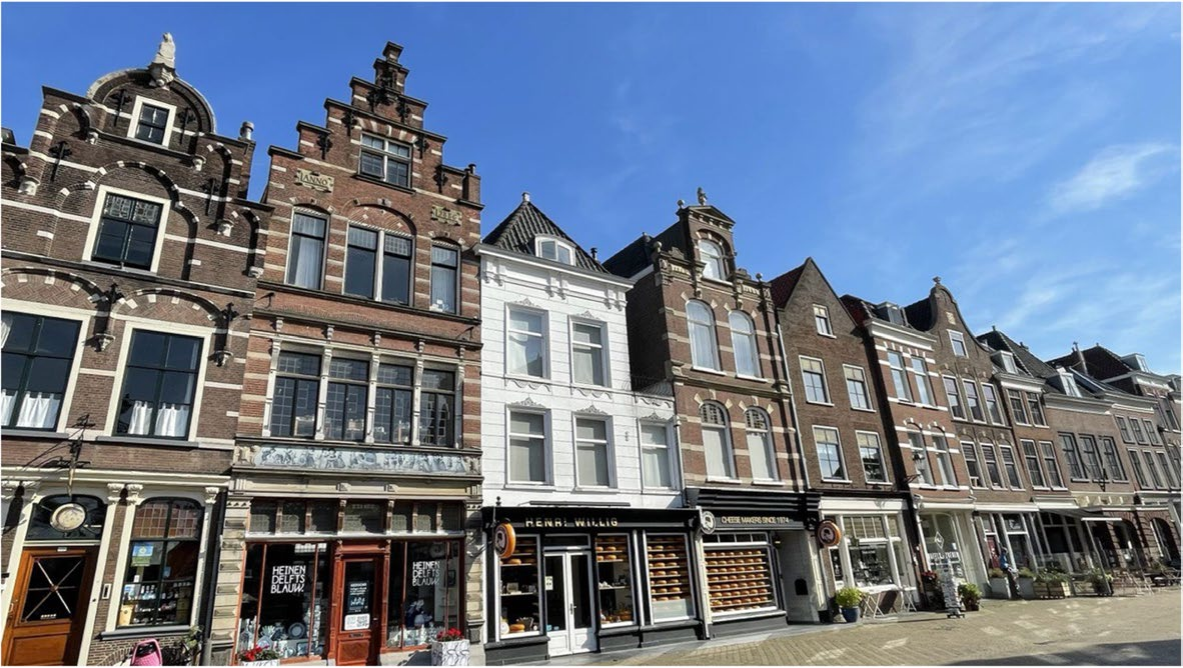
Examples of crow-stepped gable roofs in Delft

The BCGA addon already allows the automatic generation of different types of roofs: flat roofs, hip roofs, gable roofs, and mansard roofs (Fig. 9). Other roof types can easily be added to the existing implementation. Taking advantage of this, we added a rule to automatically reconstruct crow-stepped gable roofs, which are historical roofs specific to the Netherlands (Fig. 10).

The choice of the roof types for the historical 3D city model depends both on the study area and the epoch. For instance, crow-stepped gable roofs were common on traditional Dutch houses from the 12^th^ century until the beginning of the 20^th^ century, while flat roofs started to be more widely used in Northern Europe from the 19^th^ century  (Urbanik & Tomaszewicz, 2014). For more realism, building roofs are generated by varying pitches and other roof parameters. Special care must be taken to generate the street facade of the buildings on their right side (i.e. the street side). For this purpose, we use the information in the neighbours to create the street facade of a given building on the same side as its neighbours that have already been reconstructed in 3D.

With this process, the historical 3D city model is automatically reconstructed from the building footprints. Current 3D city models might be already available for the aligned building footprints. In this case, the computational workload can be reduced by reconstructing 3D buildings only for the 2PM building footprints and using the existing 3D building models for the aligned ones.

#### CityJSON generation

Without semantic information, 3D buildings are only a set of geometric primitives made of vertices and triangles. Semantic information means that a surface of a given building ‘knows’ what it is (Stadler & Kolbe, 2007); for instance, an object represented with 100 surfaces knows that it is a residential building, and a subset of these surfaces knows it is a window. As several applications of 3D city models require 3D geometries enriched with semantic information (Biljecki et al., 2015), this also holds for historical 3D city models (Hadjiprocopis et al., 2014). Hence, building faces are labelled as ground surfaces, wall surfaces and roof surfaces based on the method and tolerance values of Biljecki et al. (2016a).

The 3D buildings are represented as solids with semantic surfaces in a CityJSON file. We ensure that the geometries are valid according to the international standard ISO19107  (Ledoux, 2018) so that the files can be further processed by other software. Some software indeed expect input geometries to be free of geometric errors such as duplicated vertices, self-intersections, missing faces, etc. (Attene et al., 2013).

### Open source implementation

Our methodology solely relies on open source tools and software, and we used Python as the main programming language. In addition, we used GRASS GIS along with the GRASS Python Scripting Library to implement the method of Gobbi et al. (2019) and digitalise the historical maps (Step 1). To generalise the building footprints, we used the implementation of Commandeur (2012) (Step 1). Blender was also used for reconstructing the 3D buildings (Step 3). More particularly, we used BlenderGIS for handling geospatial data in Blender^2^, BCGA for 3D procedural modelling^3^ and *Up3date* for creating valid CityJSON files^4^. Lastly, we used the software *val3dity* (Ledoux et al., 2019) to validate the geometries (Step 3).

## Experiments with Delft and Brussels

We implemented our methodology for two different European cities, Delft and Brussels, and for different epochs in order to reconstruct dynamic historical 3D city models. We reconstructed 3D city models of Delft in 1880, 1915, 1961 and 1982, and of Brussels in 1700, 1890 and 1924. We report on the results of these experiments in the following sections.

### Real-world historical datasets used as input

**Fig. 11:**
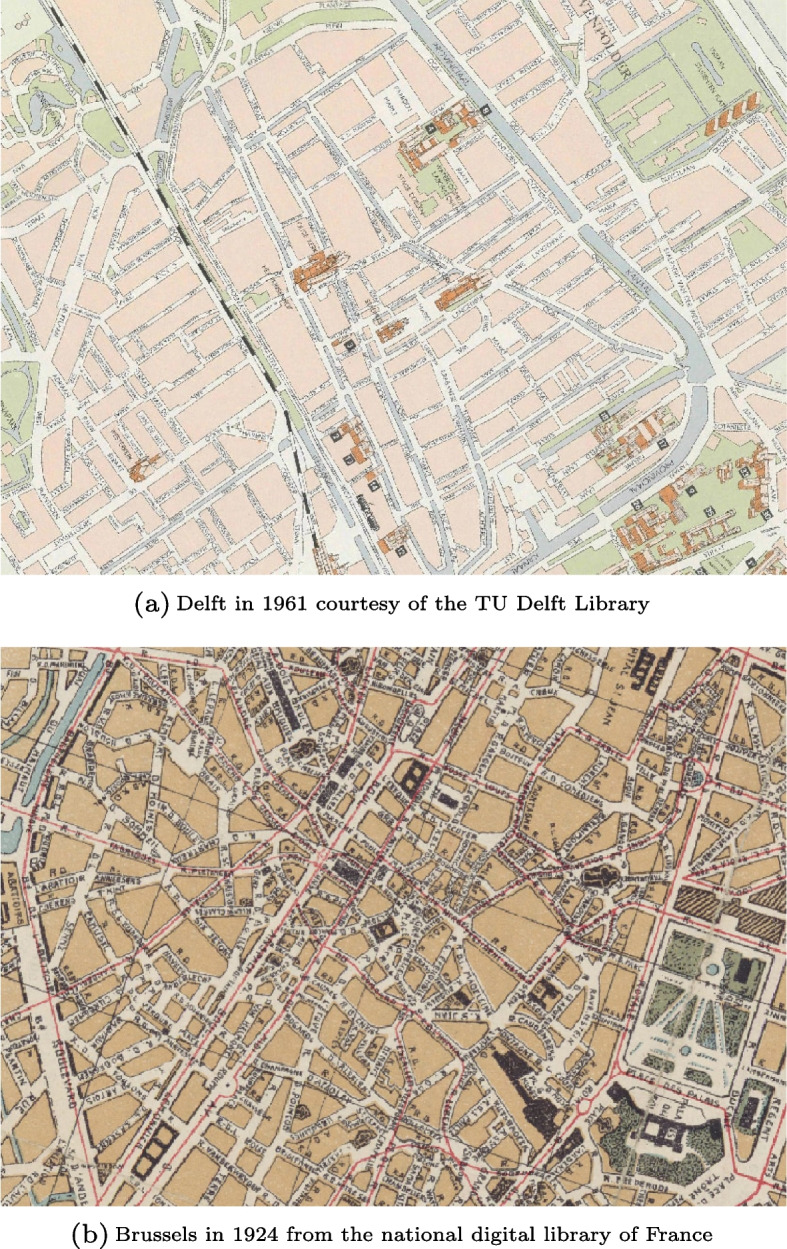
Historical maps of Delft and Brussels

Historical maps are the primary input of the methodology. In this study, we used historical maps of Delft coming from the Kadaster collection^5^ and from the TU Delft Library^6^. We also used historical maps of Brussels coming from Gallica, the national digital library of France^7^. Fig. 11 shows examples of historical maps that we used in this study. It represents the city of Delft in 1961 and Brussels in 1924.

If available, building footprint datasets enriched with the roof height and the year of construction of the buildings can be used to implement the map alignment (Step 2). For Delft, we used the *Basisregistratie Adressen en Gebouwen* (BAG) (in Dutch), which is a register containing the location information and several attributes about all buildings in the Netherlands. We also used the 3D BAG^8^ to recover 3D building models of remaining historic buildings identified by the map alignment. All 10 million buildings in the Netherlands have been automatically reconstructed, with roof structures and dormers, with the algorithm described in Peter et al. (2022). Such datasets were not available for Brussels, and we therefore did not use the map alignment.

3D point cloud datasets are needed for inferring the ground height of the building footprints. We used for Delft the *Actueel Hoogtebestand Nederland 3* (AHN3)^9^ (in Dutch). This dataset contains accurate elevation data for the Netherlands in the form of 3D points classified into different categories (e.g. ground, buildings, water, etc.). These points were acquired using LiDAR technology (Mallet & Bretar, 2009), which measures the distance to an object by sending a laser pulse and measuring the reflected signal. For Brussels, we generated a 3D point cloud by sampling the centre of the pixels of a gridded digital elevation model (DEM)^10^, which was the only elevation dataset available for the area.

### Step 1: Building plot extraction

The performance of the building plot extraction was assessed using the F-score metric, which measures both the precision and the recall (Sun et al., 2020). The precision represents the percentage of features classified as building plots and that are indeed building plots in the ground truth, while the recall is the percentage of ground truth building plots which were properly classified. For four out of the seven historical maps that we used in this study, the F-score was superior at 85%, which translates to high precision and recall values. For the other historical maps, our methodology was less effective, reaching F-score values around 70% and lower. Differences in F-score values are explained by two main factors: the quality of the scanning process and the cartography rules of the historical maps. This point is further discussed in Sec. 5.

As for the accuracy of the extraction of the shape of the building plots, the average difference between turning functions was comprised between 0.90 and 1.37 for all historical maps, while the average shape similarity (Samal et al., 2004) was above 95% for all maps except for one where it reached 90%. These values indicate that both the contours and the angles of the building plots were preserved during the extraction process and that their orientation and size were also properly extracted from the historical maps.

### Step 2: Individual building footprint generation

**Fig. 12:**
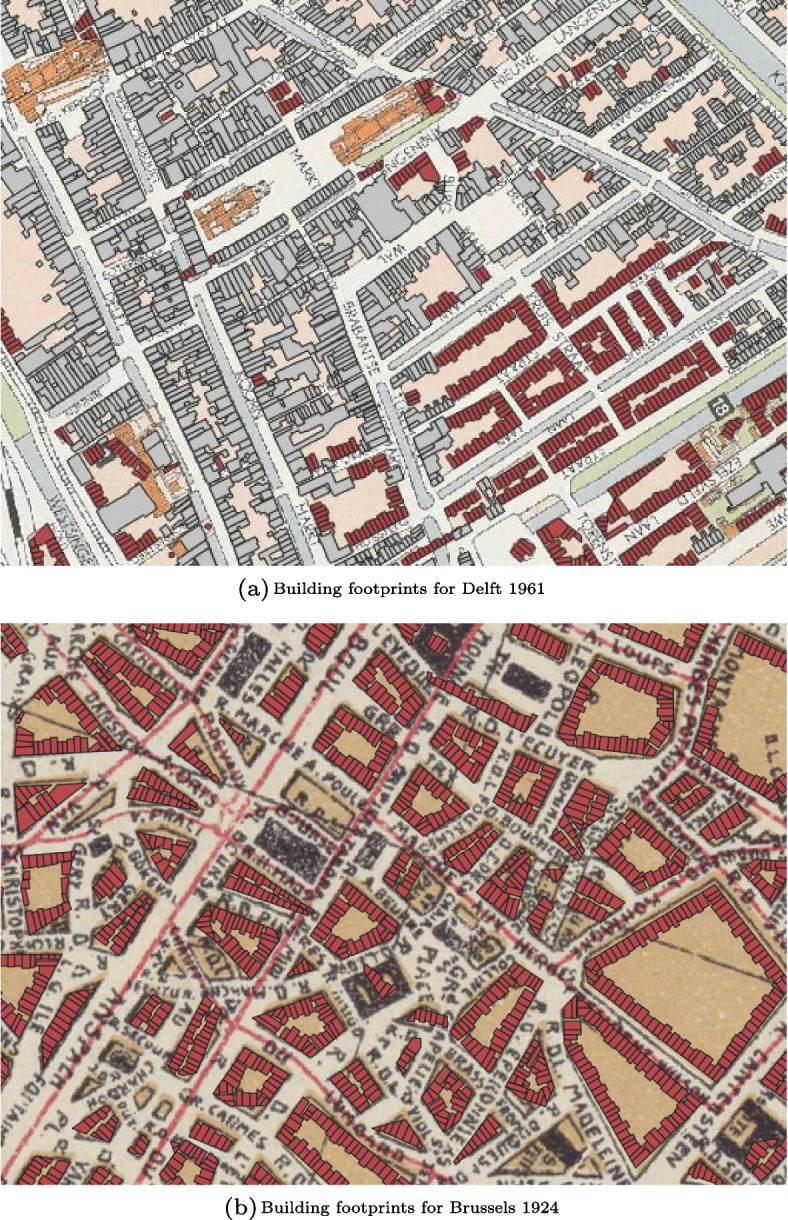
Final building footprint datasets for Delft 1961 and Brussels 1924, with aligned building footprints in grey and 2PM building footprints in red. For Brussels, the dataset only contains 2PM building footprints, as map alignment was not implemented

This step was implemented using both map alignment and 2D procedural modelling for Delft, while for Brussels, we only used 2D procedural modelling due to differences in data availability. Fig. 12 shows the building footprint datasets generated from the maps of Delft in 1961 and Brussels in 1924, where aligned building footprints are represented in grey and 2PM building footprints in red. The main difference between the two types of building footprints is that some aligned ones are non-convex, when an extension was added to the building footprint, while all 2PM building footprints are convex and thus do not have any extensions. In some cases, there is a misalignment on the street side between the aligned building footprints and the 2PM building footprints coming from the same historical building plot. This misalignment comes from an offset between the aligned building footprints and their corresponding building plot on the historical map. Different elements could explain this offset; it can be due to the quality of the georeferencing or to chronometric or geometric inaccuracies in the historical maps.

**Fig. 13:**
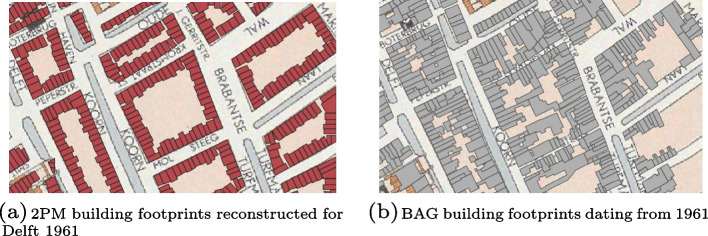
2PM building footprints (red) and their ground truth (grey) overlaid over the historical map of Delft 1961

To further assess the accuracy and realism of the 2PM building footprints, we used 2D procedural modelling to subdivide building plots from the historical map of Delft 1961 with known building footprints so that we could compare the configuration and distribution of 2PM building footprints with ground truth. The results of this experiment are shown in Fig. 13. As already highlighted in this section, the 2PM building footprints lack building extensions and are misaligned with respect to their ground truth. The 2PM building footprints however stick to the building plots on the historical map, contrarily to the ground truth which are offset with respect to them. This suggests that the misalignment comes indeed from discrepancies between the historical maps and the actual location of the buildings they depict.

### Step 3: 3D building generation

**Fig. 14:**
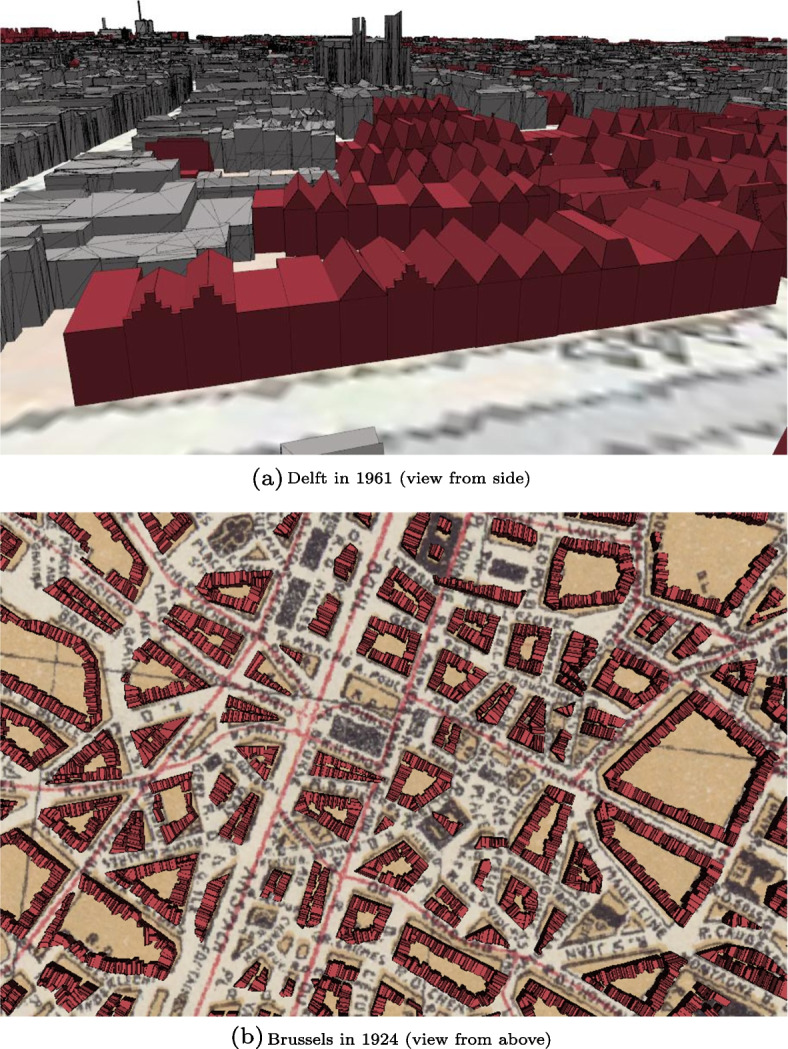
Historical 3D city models of Delft in 1961 and Brussels in 1924. Buildings depicted in red were reconstructed from 3D procedural modelling while buildings depicted in grey come from the 3D BAG (only in the case of Delft)

The last step of our methodology deals with the reconstruction of 3D buildings. Fig. 14 shows the historical 3D city models reconstructed from the historical maps of Delft 1961 and Brussels 1924 with our methodology. As the map alignment influences the implementation of step 3, we obtained different historical 3D city models for Delft and Brussels. All historical 3D city models of Brussels are made exclusively of 3D buildings generated with 3D procedural modelling, while for Delft we re-used 3D building models from the 3D BAG for the aligned building footprints. As a result, the historical 3D city models of Delft are made of two types of buildings with different levels of details. 3D building models from the 3D BAG have more complex footprints and additional building installations such as dormers or chimneys in comparison to the buildings generated with 3D procedural modelling, which always have convex footprints. Another difference between the two cities regards the assignment of the roof height; in Delft models, the roof height is based on a neighbourhood analysis using the aligned building footprints, while in Brussels it is based on regulated values coming from the literature. As a result, the roof height in Delft historical 3D city models is sometimes too high in comparison to the building height below the roof due to interpolation errors introduced by the neighbourhood analysis. Such errors happen when the roof height assigned to the 2PM building footprints is only based on one or two neighbours. If the roof height of these neighbours is not accurate or if one of them is a chapel, then these values are still transferred to the surrounding 2PM building footprints, which may hence be assigned abnormally high roof heights.

The visual assessment of the different historical 3D city models showed that the semantics are properly assigned and the street facade of the buildings is built on the correct side, the street side. In addition, all historical 3D city models reconstructed with our methodology are valid against the CityJSON schema and have more than 99% of their geometries valid as assessed by *val3dity* (Ledoux et al., 2019).

## Discussion

We have presented our methodology for reconstructing historical 3D city models from historical maps, maximizing the automation process, and our implementation is available as open source code. We have tested our methodology with historical maps of Delft and Brussels at different time periods, showing the suitability of our methodology to be applied for different European cities and historical map collections. Our methodology can be extended to any city in Europe *as long as the input historical maps are multicoloured maps displaying building plots*. As implemented, our methodology takes as input maps with building plots and not footprints (because this is what was available in the historical maps we had access to); modifying the methodology so that building footprints are used would be a simple task.

For other users focusing on different European cities, the implementation of the methodology depends on data availability. If all the datasets are available, the entire methodology can be implemented, including the map alignment, such as for historical maps of Delft. In the opposite case, this step is skipped, such as for historical maps of Brussels. This difference in data availability also influences the methodology running time, which can be large depending on the size of the study area. This is essentially due to the 3D procedural modelling step, which is more time-consuming than the other parts of the methodology. Therefore, if remaining historical buildings can be recovered from an existing 3D city model using the map alignment, the processing time is significantly reduced. Furthermore, using or not the map alignment influences the way the roof height is assigned, either making use of the aligned building footprints and their height attributes or using regulated values from the literature. Using the first option might be more accurate at the individual building level as the roof height is defined using a spatial process (i.e. the neighbourhood analysis), but it might also be more prone to interpolation errors or irregularities. Besides, using the information in the neighbours supposes that a sufficient percentage of the historical building remains. When not enough buildings are remaining, the shorter methodology version (i.e. without the map alignment) might be preferred, as implementing the entire methodology would mean assigning the roof heights relying only on a few (aligned) buildings.

Two main factors influence the performance of our methodology for other study areas: the quality of the scanning process and the cartography rules of the historical maps. The quality of the scanning process influences whether defects affect the scanned historical maps, such as the aliasing and false colour effects. These two phenomena respectively lead to mixed pixels and high colour variations inside geographic features, which both influence the quality of the map digitalisation. They can greatly influence the building plot extraction, leading to lower precision and recall values. The scanning process also deals with the choice of a spatial resolution for scanning the historical map, and this parameter highly influences the results obtained. A higher spatial resolution means more details in the contour of the geographic features and smoother transitions between pixels of different categories of features (mixed pixels). Moreover, the spatial resolution is an important factor to consider when using an object-based classification because it requires the geographic features to be large enough to be identified as objects. If the spatial resolution is too low, some geographic features may only be represented by a few pixels and are then not identified as objects. The second factor that strongly influences the results is the cartographic rules that were used to create the historical maps. For maps with strict cartographic rules, the building plot extraction performs better because all categories of geographic features are depicted in different colours. Contrarily, when different categories of features are represented using the same symbology, it is less evident for the algorithm to differentiate them.

One great advantage of our methodology is its flexibility. First, we tried in this research to find solutions around data availability, and therefore we proposed a methodology with two alternative options depending on data availability. Second, our methodology can be implemented with various collections of historical maps, while many of the existing methods that we have investigated only work for a specific map collection. However, a consequence of this is that the results are not perfect, in the sense that some errors or discrepancies are sometimes introduced. For instance, churches and other special buildings are not always recovered with this methodology as they are often represented in different ways on historical maps (e.g. symbols, 3D drawings, building plots), and thus it is difficult to implement a methodology that can recover them automatically regardless of the way they are represented. Therefore, in certain cases, these buildings should be manually added. The amount of manual processing that could be implemented to have the most accurate results depends on the input historical maps. If the map was not properly scanned, more manual work will be required for compensating the low recall and precision values. On the contrary, high recall and precision values would require almost no manual processing.

**Fig. 15:**
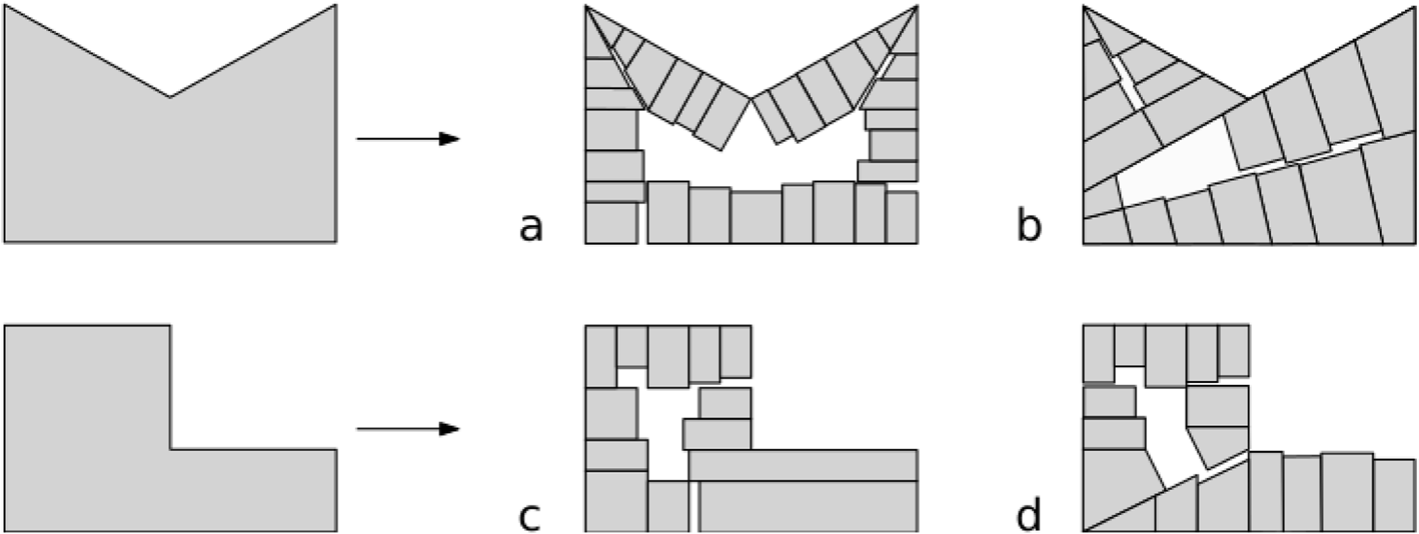
Subdivision of concave building plots into building footprints. Left-sided building footprints (a and c) have been created without using case 5 while the right-sided ones (b and d) have been generated using it. In a, the resulting building footprints look more realistic than in b and the opposite is observed in c and d

Another consequence of that flexibility is that our methodology relies on user-defined parameters. This is sometimes considered as a roadblock towards automation as it might require lots of trial and error work for finding the most suitable values. However, based on our experiments with different collections of historical maps, we provide recommendations for an optimal choice of values for these parameters. Besides, as our methodology is implemented in different steps, users wanting to adjust these values can proceed step by step, which makes it easier to identify the most suitable values. Our methodology also relies on a series of thoughtful assumptions made in order to reconstruct plausible buildings, as we are limited by the amount of historical information available. These assumptions might fail in specific cases or for certain regions. In particular, the 2D procedural modelling algorithm is based on a set of predefined rules, which might not always hold. First, the algorithm subdivides building plots into terraced houses, which might less suit peri-urban areas organized in detached houses/villas. Also, buildings are created with an alignment on the street side, although neighbouring buildings are sometimes built at varying distances from the roads. Specific to case 4 (Fig. 8), certain cities might not be characterised by an arrangement of the buildings around an interior courtyard, e.g. North American cities. As for case 5 (Fig. 8), in certain cases the decomposition of concave building plots into convex polygons before being further processed gives less realistic results than treating them directly with cases 1 to 4 without convex decomposition, see Fig. 15 for an example. The reconstruction of 3D buildings is also based on a series of assumptions. As already discussed, assigning the building height using the median height in the neighbours might fail in cases where special buildings are found. For example, if chapels or churches are part of the neighbours, they might level up the median height. We also make assumptions about the types of roofs common in the different regions. This should be adapted given the city. For example, crow-stepped gable roofs are less common in Brussels than in Delft and completely absent from certain cities, e.g. African cities.

Finally, with this work, we provide a methodology that allows the reconstruction of a historical 3D city model, with limited user intervention, that probably looks like what the city looked like at a given time. We reconstruct multipurpose LoD2 building models that can be useful for disease mapping (through population estimation), visibility analyses and other spatial analyses. Although our models are not perfect and are affected by discrepancies (as already discussed with specific buildings and the misalignment of 2PM building footprints), we argue that they are a good mean to automate the reconstruction process, while reconstructing an historical 3D city model manually would be very time and money-consuming. Hence, our models supplement the manually made ones when time and money are limited, in two main ways. First, they can act as a starting point; one could start from our automated model and further modify it by simply replacing the 3D buildings of its choice (which is easily done as we use semantic 3D city models). It is also possible to automatically add façade details (e.g. windows, doors, and balconies) to simple building models like ours, see Zhang et al. (2019) and Wang (2022) for two examples. The only auxiliary dataset necessary is street-view imagery (Biljecki & Ito, 2021), which, while not always widely available for the past, is publicly accessible in some counties: in the Netherlands there are several thousands images available for instance^11^. Second, they can be complementary to the manually made models by enabling the automatic processing of some parts of the input historical map while keeping manual the reconstruction of more important areas. For instance, one could imagine reconstructing manually the city centre and automating the reconstruction of the outskirts. Also, our method could be used to reconstruct LoD1 building models (i.e. with flat roofs) over which more detailed roofs could be further manually added to create LoD2 buildings. Automating some parts of the reconstruction process will become even more advantageous when several historical 3D city models are needed for time series, for instance.

## Future work

Some parts of our methodology could benefit from additional focus to improve the general workflow. Our methodology could be improved by making it possible to create buildings with non-convex footprints with 2D procedural modelling. The BCGA addon algorithm should then also be extended by adding rules for reconstructing cross hip/gable roofs (Fig. 9).

Besides improving our methodology, there are also some areas that we did not explore and that could be interesting to investigate in the future. One such area is the use of other types of historical sources in addition to historical maps, which could bring added value to the historical 3D city models reconstructed. For instance, the actual building height could be extracted from historical aerial images using machine learning techniques  (Lánský, 2020), or from historical photographs/postcards (Kersten et al., 2012). Furthermore, it would also be interesting to investigate the reconstruction of other types of features than buildings such as canals, roads, and trees, as it could also lead to interesting applications. For instance, in medical mapping, the reconstruction of canals and rivers could provide additional insights into the propagation of waterborne diseases (Bidhuri & Jain, 2019).

## Data Availability

The datasets used as input, and generated during by your methodology, are available in the following repository: https://github.com/CamilleMorlighem/histo3d-data.

## Footnotes

1 Data and code are respectively available at https://github.com/CamilleMorlighem/histo3d-data and https://github.com/camilleMorlighem/histo3d

2 https://github.com/domlysz/BlenderGIS

3 https://github.com/vvoovv/bcga

4 https://github.com/cityjson/Up3date

5 https://www.topotijdreis.nl/

6 https://heritage.tudelft.nl/en/collections/lib-kaartenkamer

7 https://gallica.bnf.fr/

8 https://3dbag.nl/

9 https://downloads.pdok.nl/ahn3-downloadpage/

10 https://datastore.brussels

11 https://beeldbank.cultureelerfgoed.nl/

## Code availability

The full code of the methodology is open source: https://github.com/camilleMorlighem/histo3d.

## References

[CR1] Arkin, E. M., Chew, L. P., Huttenlocher, D. P., Kedem, K., & Mitchell, J. S. B. (1991). An efficiently computable metric for comparing polygonal shapes. *IEEE Transactions on Pattern Analysis and Machine Intelligence,**13*(3), 209–216. 10.1109/34.75509.

[CR2] Arteaga, M. G. (2013). Historical Map Polygon and Feature Extractor. In *MapInteract ’13: Proceedings of the 1st ACM SIGSPATIAL International Workshop on MapInteraction* (pp 66–71). New York. 10.1145/2534931.2534932

[CR3] Attene, M., Campen, M., & Kobbelt, L. (2013). Polygon mesh repairing: An application perspective. *ACM Computing Surveys,**45*(2), 15:1-15:33. 10.1145/2431211.2431214.

[CR4] Balletti, C., & Guerra, F. (2016). Historical Maps for 3D Digital Cities History. *Cartographica: The International Journal for Geographic Information and Geovisualization,**51*(3), 115–126. 10.3138/CART.51.3.3140.

[CR5] Bayazit, M. (2009). Mark bayazit’s algorithm. https://mpen.ca/406/bayazit. Accessed 25 Oct 2021

[CR6] Bidhuri, S., & Jain, P. (2019). Identifying waterborne disease prone areas using geospatial approach along the right bank of Yamuna River in Delhi. *International journal of environmental health research,**29*(5), 561–581. 10.1080/09603123.2018.1557121. 10.1080/09603123.2018.1557121 30569747

[CR7] Biljecki, F., & Ito, K. (2021). Street view imagery in urban analytics and GIS: A review. *Landscape and Urban Planning,**215*. 10.1016/j.landurbplan.2021.104217.

[CR8] Biljecki, F., Ledoux, H., Du, X., Stoter, J., Soon, K., & Khoo, V. (2016). The most common geometric and semantic errors in CityGML datasets. *ISPRS Annals of the Photogrammetry, Remote Sensing and Spatial, Information Sciences,**IV–2/W1,* 13–22. 10.5194/isprs-annals-IV-2-W1-13-2016.

[CR9] Biljecki, F., Ledoux, H., & Stoter, J. (2016). An improved LODspecification for 3D building models. *Computers, Environment and Urban Systems,**59,* 25–37. 10.1016/j.compenvurbsys.2016.04.005.

[CR10] Biljecki, F., Stoter, J., Ledoux, H., Zlatanova, S., & Çöltekin, A. (2015). Applications of 3D city models: State of the art review. *ISPRS International Journal of Geo-Information,**4*(4), 2842–2889. 10.3390/ijgi4042842.

[CR11] Commandeur, T. (2012). Footprint decomposition combined with point cloud segmentation for producing valid 3D models. Master’s thesis, MSc Geomatics. Delft University of Technology. http://resolver.tudelft.nl/uuid:c0c665f7-0254-42c6-895b-cb59acc079f2

[CR12] de Boer A (2010). Processing old maps and drawings to create virtual historic landscapes. e-Perimetron.

[CR13] Douglas, D. H., & Peucker, T. (1973). Algorithms for the reduction of the number of points required to represent a digitized line or its caricature. *Cartographica: The International Journal for Geographic Information and Geovisualization,**10*(2), 112–122. 10.3138/FM57-6770-U75U-7727.

[CR14] Drolias, G. C., & Tziokas, N. (2020). Building Footprint Extraction from Historic Maps utilizing Automatic Vectorisation Methods in Open Source GIS Software. *International workshop on automatic vectorisation of historical maps*. Budapest: ELTE. 10.21862/avhm2020.01.

[CR15] Dukai, B., Ledoux, H., & Stoter, J. (2019). A multi-height LoD1 model of all buildings in the Netherlands. *ISPRS Annals of Photogrammetry, Remote Sensing and Spatial Information Sciences,**IV–4/W8,* 51–57. 10.5194/isprs-annals-IV-4-W8-51-2019.

[CR16] Fan, H., Zipf, A., Fu, Q., & Neis, P. (2014). Quality assessment for building footprints data on OpenStreetMap. *International Journal of Geographical Information Science,**28*(4), 700–719. 10.1080/13658816.2013.867495.

[CR17] Frischer, B., Abernathy, D., Guidi, G., Myers, J., Thibodeau, C., Salvemini, A., Müller, P., Hofstee, H., & Minor, B. (2008). Rome Reborn. In *International Conference on Computer Graphics and Interactive Techniques* (p 34). Los Angeles. 10.1145/1401615.1401649

[CR18] Galanaud, P., Galanaud, A., & Giraudoux, P. (2015). Historical Epidemics Cartography Generated by Spatial Analysis: Mapping the Heterogeneity of Three Medieval “Plagues” in Dijon. *PLoS ONE,**10*(12). 10.1371/journal.pone.0143866. 10.1371/journal.pone.0143866 PMC4666600 26625117

[CR19] Gobbi S, Ciolli M, La Porta N, Rocchini D, Tattoni C, Zatelli P (2019). New Tools for the Classification and Filtering of Historical Maps. ISPRS International Journal of Geo-Information.

[CR20] Hadjiprocopis, A., Ioannides, M., Wenzel, K., Rothermel, M., Johnsons, P. S., Fritsch, D., Doulamis, A. D., Protopapadakis, E., Kyriakaki, G., Makantasis, K., Weinlinger, G., Klein, M., Fellner, D., Stork, A., & Santos, P. (2014). 4D reconstruction of the past: the image retrieval and 3D model construction pipeline. In *Proceedings of SPIE - The International Society for Optical Engineering* vol. 9229. 10.1117/12.2065950

[CR21] Held M, Palfrader P (2017). Straight skeletons with additive and multiplicative weights and their application to the algorithmic generation of roofs and terrains. Computer-Aided Design.

[CR22] Henderson, T., Linton, T., Potupchik, S., & Ostanin, A. (2009). 8^th^ IAPR International Workshop on Graphics Recognition - GREC 2009. *Automatic Segmentation of Semantic Classes in Raster Map Images*. France: La Rochelle.

[CR23] Herrault, P.-A., Sheeren, D., Fauvel, M., Monteil, C., & Paegelow, M. (2013). A comparative study of geometric transformation models for the historical ‘Map of France’ registration. *Geographia Technica,**17*(1), 34–46.

[CR24] Herrault, P.-A., Sheeren, D., Fauvel, M., & Paegelow, M. (2013). Automatic Extraction of Forests from Historical Maps Based on Unsupervised Classification in the CIELab Color Space. In D. Vandenbroucke, B. Bucher, & J. Crompvoets (Eds.), *Geographic information science at the heart of europe. lecture notes in geoinformation and cartography.* New York. 10.1007/978-3-319-00615-4_6

[CR25] Kersten, T. P., Keller, F., Saenger, J., & Schiewe, J. (2012). Automated Generation of an Historic 4D City Model of Hamburg and Its Visualisation with the GE Engine. In *Proceedings of the 4th International Conference on Progress in Cultural Heritage Preservation* (vol. 7616, pp 55–65). Limassol. 10.1007/978-3-642-34234-9_6

[CR26] Ketzler B, Naserentin V, Latino F, Zangelidis C, Thuvander L, Logg A (2020). Digital Twins for Cities: A State of the Art Review. Built Environment.

[CR27] Lánský, I. (2020). Height inference for all USA building footprints in the absence of height data. Master’s thesis, MSc Geomatics. Delft University of Technology. http://resolver.tudelft.nl/uuid:ddcae7d1-6cc8-42a7-8c1d-a922ec7551f0

[CR28] Ledoux H (2018). val3dity: validation of 3D GIS primitives according to the international standards. Open Geospatial Data, Software and Standards.

[CR29] Ledoux H, Ohori KA, Kumar K, Dukai B, Labetski A, Vitalis S (2019). CityJSON: a compact and easy-to-use encoding of the CityGML data model. Open Geospatial Data, Software and Standards.

[CR30] Liu T, Xu P, Zhang S (2019). A review of recent advances in scanned topographic map processing. Neurocomputing.

[CR31] Mallet C, Bretar F (2009). Full-waveform topographic lidar: State-of-the-art. ISPRS Journal of Photogrammetry and Remote Sensing.

[CR32] Nobajas A, Nadal F (2015). From historical map to online 3D recreation: the 1861 cadastral map of Horta (Barcelona). Cartography and Geographic Information Science.

[CR33] Pađen, I., García-Sánchez, C., & Ledoux, H. (2022). Towards automatic reconstruction of 3D city models tailored for urban flow simulations. *Frontiers in Built Environment*, 8(899332)

[CR34] Parish, Y. I. H. & Müller, P. (2001). Procedural Modeling of Cities. In *Proceedings of the 28*^*th*^*Annual Conference on Computer Graphics and Interactive Techniques* (pp 301–308). New York. 10.1145/383259.383292

[CR35] Peters, R., Dukai, B., Vitalis, S., van Liempt, J., & Stoter, J. (2022). Automated 3D reconstruction of LoD2 and LoD1 models for all 10 million buildings of the Netherlands. *Photogrammetric Engineering and Remote Sensing*, 88(3), 165–170

[CR36] Samal, A., Seth, S., & Cueto1, K. (2004). A feature-based approach to conflation of geospatial sources. *International Journal of Geographical Information Science,**18*(5), 459–489. 10.1080/13658810410001658076

[CR37] Stadler, A. & Kolbe, T. H. (2007). Spatio-semantic coherence in the integration of 3D city models. In Stein, A., editor, *International Archives of Photogrammetry, Remote Sensing and Spatial Information Sciences. Proceedings of the WG II/7 5*^*th*^*International Symposium Spatial Data Quality 2007 with the theme: Modelling qualities in space and time* (p 8). Enschede, the Netherlands

[CR38] Sun, K., Hu, Y., Song, J., & Zhu, Y. (2020). Aligning geographic entities from historical maps for building knowledge graphs. *International Journal of Geographical Information Science,**35*(10), 2078–2107. 10.1080/13658816.2020.1845702.

[CR39] Suzuki, S. & Chikatsu, H. (2003). Recreating the past city model of historical town Kawagoe from antique map. *International Archives of the Photogrammetry, Remote Sensing and Spatial Information Sciences*, XXXIV-5/W10

[CR40] Urbanik, J. & Tomaszewicz, A. (2014). Flat Roof - Advantage or Disadvantage of Modern Movement Buildings. In *SAHC 2014: 9*^*th*^* International Conference on Structural Analysis of Historical Constructions*, Mexico City

[CR41] Wang, L. (2022). Detailed Facade Reconstruction for Mahattan-world Buildings. Master’s thesis, MSc Geomatics. Delft University of Technology. http://resolver.tudelft.nl/uuid:c0c665f7-0254-42c6-895b-cb59acc079f2

[CR42] Xavier, E. M. A., Ariza-López, F. J., & Ureña Cámara, M. A. (2016). A Survey of Measures and Methods for Matching Geospatial Vector Datasets. *ACM Computing Surveys,**49*(2), 1–34. 10.1145/2963147.

[CR43] Zhang, X., Lippoldt, F., Chen, K., Johan, H., & Erdt, M. (2019). A data-driven approach for adding facade details to textured LoD2 CityGML models. In *Proceedings 14*^*th*^* International Joint Conference on Computer Vision, Imaging and Computer Graphics Theory and Applications* (pp 294–301). 10.5220/0007507802940301

